# Supplementary material for: Reliability and Quality of YouTube Videos on Ultrasound-Guided Brachial Plexus Block: A Programmatical Review
Source: Healthcare (Basel). 2021 Aug 23;9(8):1083. doi: 10.3390/healthcare9081083 (PMC8394722; doi:10.3390/healthcare9081083)
Supplement: Supplementary file 1 [file healthcare-09-01083-s001.zip › Table S1.pdf]

Supplementary Materials

Table S1: The Journal of American Medical Association Score Benchmark Criteria

**The Journal of American Medical Association Benchmark Criteria**	
**Authorship**	Authors and contributors, their affiliations, and relevant credentials should be provided.
**Attribution**	References and sources for all content should be listed clearly, and all relevant copyright information noted.
**Disclosure**	Web site "ownership" should be prominently and fully disclosed, as should any sponsorship, advertising, underwriting, commercial funding arrangements or support, or potential conflicts of interest.
**Currency**	Dates that content was posted and updated should be indicated.
