# Supplementary material for: Reliability and Quality of YouTube Videos on Ultrasound-Guided Brachial Plexus Block: A Programmatical Review
Source: Healthcare (Basel). 2021 Aug 23;9(8):1083. doi: 10.3390/healthcare9081083 (PMC8394722; doi:10.3390/healthcare9081083)
Supplement: Supplementary file 1 [file healthcare-09-01083-s001.zip › Table S2.pdf]

Supplementary Materials

Table S2: The Global Quality Score

**Global Quality Score**	
**1**	Poor quality; very unlikely to be of any use to patients
**2**	Poor quality but some information present; of very limited use to patients
**3**	Suboptimal flow, some information covered but important topics missing; somewhat useful to patients
**4**	Good quality and flow, most important topics covered; useful to patients
**5**	Excellent quality and flow; highly useful to patients
