# Supplementary figures and images for: Reliability and Quality of YouTube Videos on Ultrasound-Guided Brachial Plexus Block: A Programmatical Review
Source: Healthcare (Basel). 2021 Aug 23;9(8):1083. doi: 10.3390/healthcare9081083 (PMC8394722; doi:10.3390/healthcare9081083)

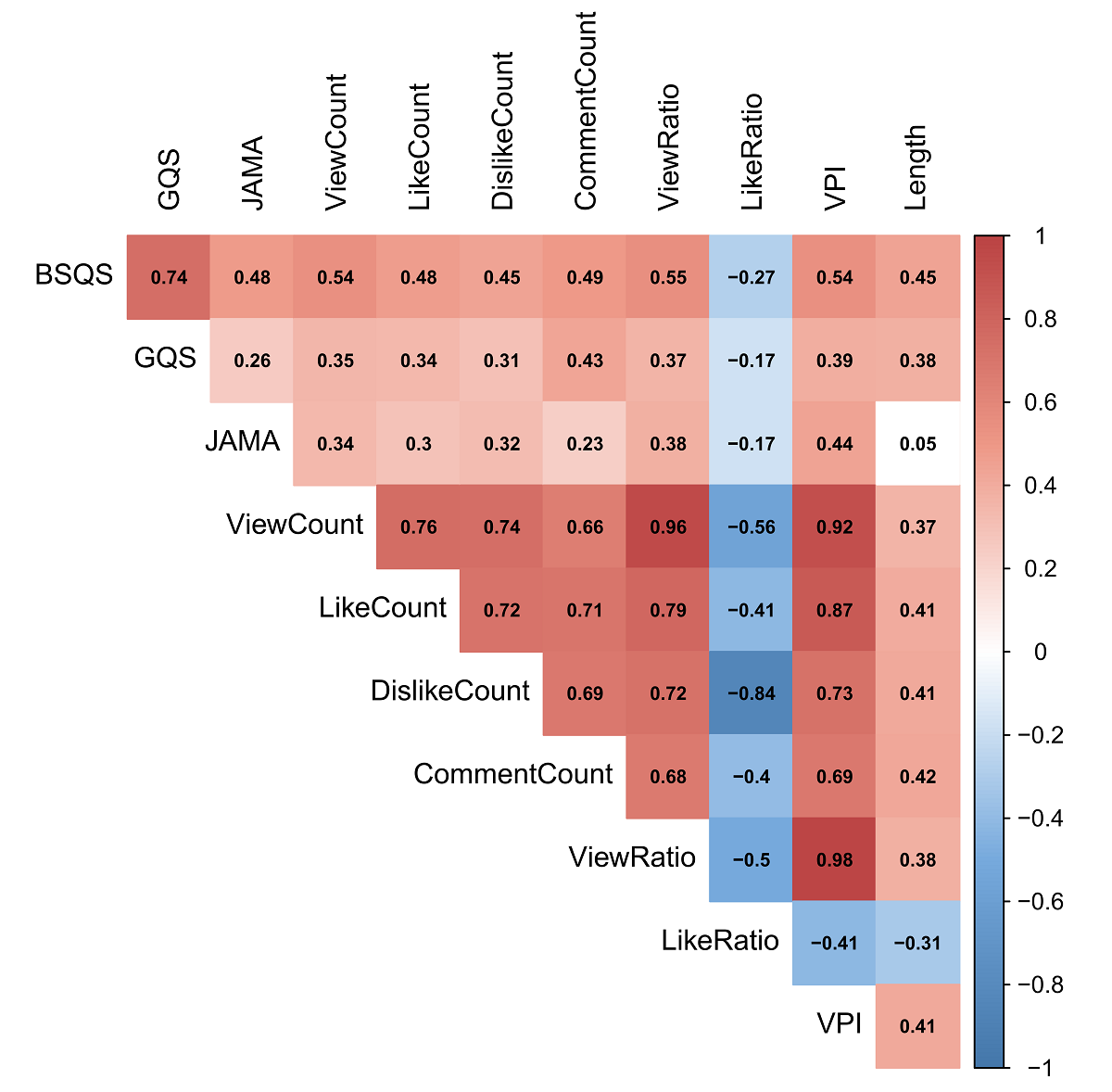

Supplement: Supplementary file 1 [file healthcare-09-01083-s001.zip › Figure S1.png]
